# Evaluation of Properties and Microstructure of Cement Paste Blended with Metakaolin Subjected to High Temperatures

**DOI:** 10.3390/ma12060941

**Published:** 2019-03-21

**Authors:** Wenqiang Wang, Xinhao Liu, Liang Guo, Ping Duan

**Affiliations:** 1 Engineering Research Center of Nano-Geomaterials of Ministry of Education, Faculty of Materials Science and Chemistry, China University of Geosciences, Wuhan 430074, China; 13125153241@163.com (W.W.); 15071242653@163.com (X.L.); liang_guo@cug.edu.cn (L.G.); 2 State Key Laboratory of Silicate Materials for Architectures, Wuhan University of Technology, Wuhan 430070, China

**Keywords:** mechanical properties, microstructure, metakaolin, cement, high temperature

## Abstract

The effects of 10% metakaolin addition on compressive strength, water absorption, shrinkage and microstructure evolution of cement paste after elevated temperatures exposure from room temperature to 800 °C were evaluated. The experimental results show that compressive strength increases at 200 °C and 400 °C compared with that obtained at ambient temperature. Up to 800 °C, compressive strength decreases rapidly. The addition of 10% metakaolin leads to the enhancement of compressive strength regardless of exposure temperatures. After thermal exposure at 400 °C, compressive strength reaches the maximum value. Thermal exposure degrades pore structure. A polynomial equation was used to indicate the shrinkage of cement paste or metakaolin-blended cement paste with testing days. Mechanical properties, permeability resistance, and shrinkage in cement pastes are closely related to the microstructure development. 10% metakaolin addition presents better thermal resistance, lower shrinkage and denser microstructure compared with pure cement paste before and after thermal exposure.

## 1. Introduction

Metakaolin (MK) is produced by calcination of kaolin at the temperatures of 500–800 °C [1,2]. Calcination was used for the purpose of producing metakaolin with high chemical reactivity for usage in cement or concrete as supplementary cementitious material [3,4,5,6]. 

Partial replacement of Portland cement by SCMs such as metakaolin represents a common route to reduce CO_2_ emissions and increase mechanical properties [7]. Extensive research efforts have been carried out to focus on the mechanical properties, pore structure and durability aspects of ordinary Portland cement blended with metakaolin.

According to previous research [8,9,10,11,12], high reactivity metakaolin has been used for more durable concrete production and it has already been proved that replacement of cement by metakaolin led to higher early strength, lower porosity, denser microstructure and better chemical corrosion resistance.

Furthermore, in a severe service environment such as seawater, a cold area or areas with great day–night temperature difference, metakaolin was also widely used to impart an additional performance to concrete [13]. 

The concrete elements are subjected to the high temperatures when in fire, which may result in significant losses of load-bearing capacity due to the reduction of a material’s strength and stiffness [14]. In terms of the properties of cement or concrete exposed to high temperature, relevant work has been widely carried out. Khaliq et al. [15] investigated strength of calcium aluminate cement concrete (CACC) from room temperature to 800 °C, and made comparison with conventional normal strength concrete. They concluded that the presence of alumina had an improvement effect on compressive strength when compared with normal strength concrete. Maarup et al. [16] explored the flowability of cement raw meal at temperatures up to 850 °C, and they reported that the flowability was reduced with temperature over 550 °C due to increasing unconfined yield strength and reduced flowability factors. Donatello et al. [17] presented properties and microstructure evolution of cement paste incorporating fly ash when exposed to 1000 °C. Higher residual strengths after heat exposure were observed in fly ash blended cement paste, and the residual strength exhibited continual decrease after thermal cycles. Ergüna et al. [18] assessed the influences of heat exposure and cement content on the compressive strength of concrete exposed to thermal cycles with different target temperatures. They concluded that concrete suffered notable compressive strength loss after being exposed to a thermal cycle with target temperature of 400 °C when compared with the reference group. Furthermore, dielectric characterization of dry concrete after thermal cycles with target temperature of 700 °C was reported by Soldatov et al. [19]. They concluded that permittivity change correlated with moisture loss and thermal decomposition reactions with increasing temperature. 

It has been realized that after collecting the previously reported results, there is a lack of experimental data on the comparison between cement paste and cement–metakaolin paste after exposed to elevated temperatures. Only Rashad et al. [20] investigated compressive strength of cement-ground granulated blast furnace slag (GGBS) paste modified with metakaolin subjected to elevated temperatures. Bu et al. [21] carried out research to assess the strength of oil well cement blended with metakaolin. Subasi et al. [22] also concluded that metakaolin replacement increased the strength due to the pozzolanic effects. Limited efforts have been made to compare the influences of metakaolin-blended cement paste with neat cement paste after thermal exposure. Compressive strength and the microstructure change of cement paste with the addition of metakaolin subjected to thermal exposure have not been revealed. The effects of metakaolin addition on high temperature properties including strength, shrinkage and water absorption of cement paste still requires further investigation.

In this work, the effects of 10% metakaolin addition on the compressive strength, water absorption resistance, shrinkage and microstructure evolution of cement paste were investigated and assessed. A comparison was made to evaluate the property changes before and after elevated temperatures exposure from room temperature to 800 °C.

## 2. Experimental

### 2.1. Materials

Ordinary Portland cement (P·I 52.5) is used according to the Chinese National Standard GB/T 175-2007. The MK is obtained from calcination of a kaolinitic mineral sourced by Maoming Kaolin Technology Co. (Maoming, China) at 750 °C for 2 h. The chemical compositions of the starting materials are listed in Table 1. The physical properties of the raw cement are given in Table 2. A polycarboxylic-ether type superplasticizer (SP) is employed to achieve the designed workability for the cement pastes.

**Figure 1:**
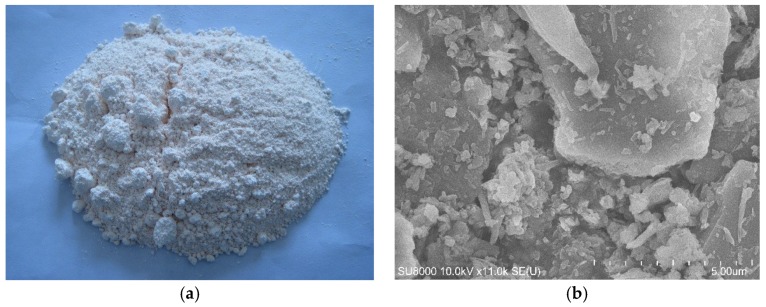
Scanning electron micrographs (SEM) of metakaolin. (**a**) Appearance; (**b**) microstructure.

The micrograph of metakaolin is shown in Figure 1. It can be noted that metakaolin indicates irregular and sheet shape. It also displays the presence of fine particles being perceived as an agglomeration of particles.

### 2.2. Specimen Preparation

Two series of pastes are prepared. The mixture proportions details are given in Table 3. 

**Figure 2:**
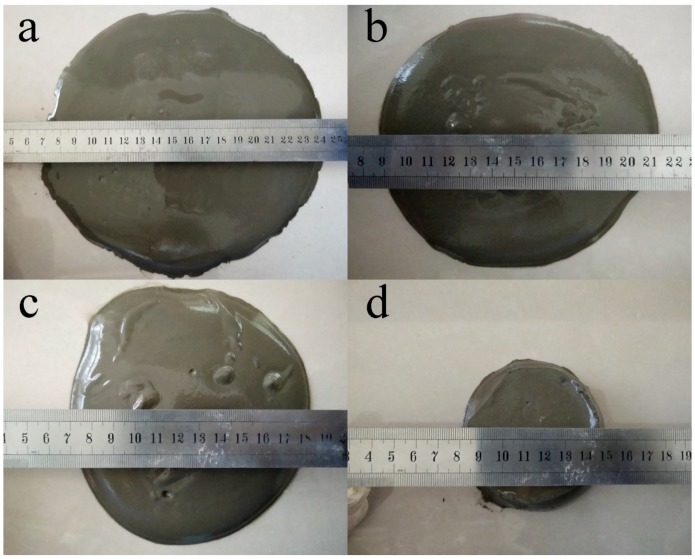
The flowability of cement paste with various replacement level of metakaolin: (**a**) 0; (**b**) 5%; (**c**) 10%; (**d**) 20%.

As Figure 2 shows, 10% replacement level was selected based on the reasonable workability consideration due to the high water acquirement of metakaolin with large specific surface area. More than 10% metakaolin addition reduces the workability significantly and affects the compacting and casting, which is due to the high reactivity of MK, very high specific surface area and its amorphous structure [23]. We tried to add different amounts of metakaolin into cement and found that the workability of fresh cement paste with the same w/b ratio reduced after adding metakaolin and the paste with 20% metakaolin had almost no fluidity as indicated in Figure 2. A 10% replacement level of metakaolin in cement was selected based on the reasonable workability consideration due to the high water acquirement of metakaolin with large specific surface area. More than 10% metakaolin addition reduces the workability significantly and affects the compacting and casting.

All the pastes were mixed in a revolving mixer. Specimens with the dimension of 40 × 40 × 40 mm^3^ were cast in steel moulds and compacted on a vibrating table. The specimens were released from the moulds 24 h after casting, and were subjected to curing in standard condition of 20 °C and 95% relative humidity (RH) up to 28 days.

At the end of the curing period, all the specimens were dried at 50 °C for 24 h and were exposed to high temperatures at 200 °C, 400 °C, 600 °C and 800 °C, respectively. The specimens were kept in the furnace for 2 h under each temperature. The heating rate of the furnace was selected as 3.0 °C/min [24]. After that, all the specimens were allowed to cool naturally to ambient temperature inside the furnace.

### 2.3. Characterization Methods

#### 2.3.1. Compressive Strength

Mechanical properties tests were conducted on the hardened cement paste and metakaolin-cement paste specimens before and after the exposed high temperatures. High temperature strength was evaluated by measuring the compressive strength, which were left for cooling at room temperature naturally for one day after being exposed to high temperature. Compressive strength was tested on 40 × 40 × 40 mm^3^ cubes by using the testing machine SFK-300/10 (Dadi Huayu Instrument Equipment Co., Ltd, Beijing, China). The average values of three specimens were recorded and reported for each test result.

#### 2.3.2. Mercury Intrusion Porosimetry (MIP)

The pore structure including porosity and pore size distribution of different series of pastes including hardened cement paste and metakaolin-cement paste specimens before and after exposed high temperatures, were measured by mercury intrusion porosimetry operated on AutoPore IV 9500 series porosimeter (Micromeritics, Norcross, GA, USA). The measurable pore size in diameter ranges from about 6 nm to 360 µm. The fragment specimens in the shape of pellets with the size of 5 × 5 × 5 mm^3^ before and after elevated temperature exposure for pore structure testing were separated from the crushed specimens after strength test and were also dried at 50 °C for 1 day before the mercury intrusion porosimetry (MIP) test.

#### 2.3.3. X-ray Diffraction (XRD)

For X-ray diffraction (XRD) analysis, samples extracted from the crushed cubes including hardened cement paste and metakaolin-cement paste specimens before and after exposed high temperatures were stored in absolute ethanol (95%) for at least 3 days to stop hydration. The dried samples were ground down to pass through a 75-μm sieve to be used in a Bruker D8 Advance X-ray diffractometer (Billerica, MA, USA) using Cu Ka radiation (40 kV, 30 mA) with a scanning rate of 2° 2θ/step from 5° to 60° 2θ.

#### 2.3.4. Fourier Transform Infrared Spectroscopy (FTIR)

Fourier transform infrared (FTIR) spectra of the cement hydration products taken from hardened cement paste and metakaolin-cement paste specimens before and after being exposed to high temperatures were collected by using a Nicolet iS50 Series FTIR spectrometer (Thermo Fisher Scientific, Waltham, MA, USA) to observe the change of hydration product structure. Discs of finely powdered products-KBr mixture (1:240 products: KBr, w/w) were prepared with the aid of a hydraulic press. Spectra were recorded in the range of 4000 to 450 cm^−1^ by the co-addition of 16 scans with an 8 cm^−1^.

#### 2.3.5. Scanning Electron Microscopy (SEM)

The development of inner microstructure, textural characteristics and morphological changes of cement phases was observed by using SU8000 scanning electron microscopy (SEM, Hitachi, Tokyo, Japan), operating at an accelerating voltage of 15 kV for photomicrographs. The specimens before and after elevated temperature exposure for microstructure observation were separated from the crushed specimens including hardened cement paste and metakaolin-cement paste specimens before and after exposed high temperatures after strength test and were dried at 50 °C for 1 day before the SEM test.

#### 2.3.6. Shrinkage

After being cured for 1 day, a different series of paste specimens including hardened cement paste and metakaolin-cement paste specimens before and after being exposed to high temperatures with the size of 20 × 20 × 200 mm^3^ were prepared for a shrinkage test. The specimens were installed onto the setup for the length change tests and cured in a curing chamber with constant temperature of 20 ± 3 °C and relative humidity of 60 ± 5%. Length changes of the specimens before and after thermal cycles were recorded and reported by reading the dial gauge regularly. Variations in the shrinkage are monitored during the 28-day period and the average values of three prism specimens are reported. Measurements are carried out every 24 h for the whole testing period.

#### 2.3.7. Water Absorption

Water absorption was carried out on paste specimens with the size of 20 × 20 × 20 mm^3^ for both hardened cement paste and metakaolin-cement paste specimens before and after exposed high temperatures. Half of the specimens were dried for 24 h at 50 °C after 28 days of standard curing (specimens before heat treatment), half of specimens were dried for 24 h at 50 °C after 28 days of standard curing and then were exposed to elevated temperatures (specimens after heat treatment). After that, the cement paste and metakaolin-blended paste specimens were immersed in deionized water for 1, 3, 7, 14 and 28 days and the mass changes were recorded to assess the water permeability resistance of pastes before and after high-temperature exposure. Before immersed into water, specimens after high temperatures exposure were allowed to cool naturally to ambient temperature.

## 3. Results and Discussion

### 3.1. Compressive Strength and Appearance

**Figure 3:**
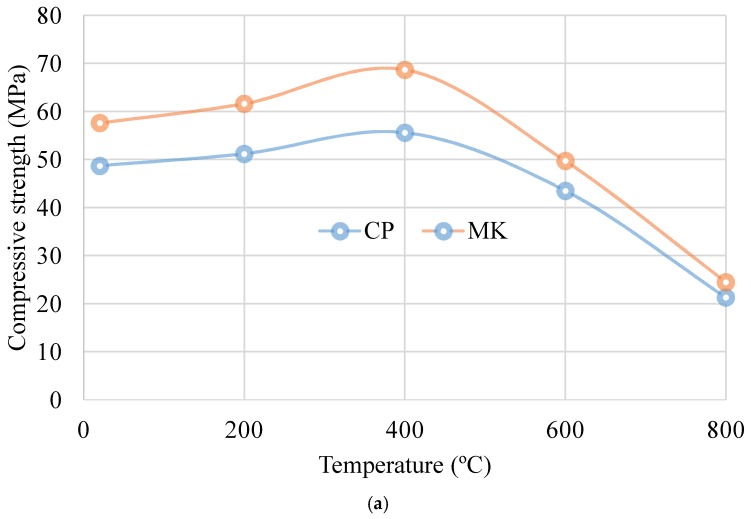

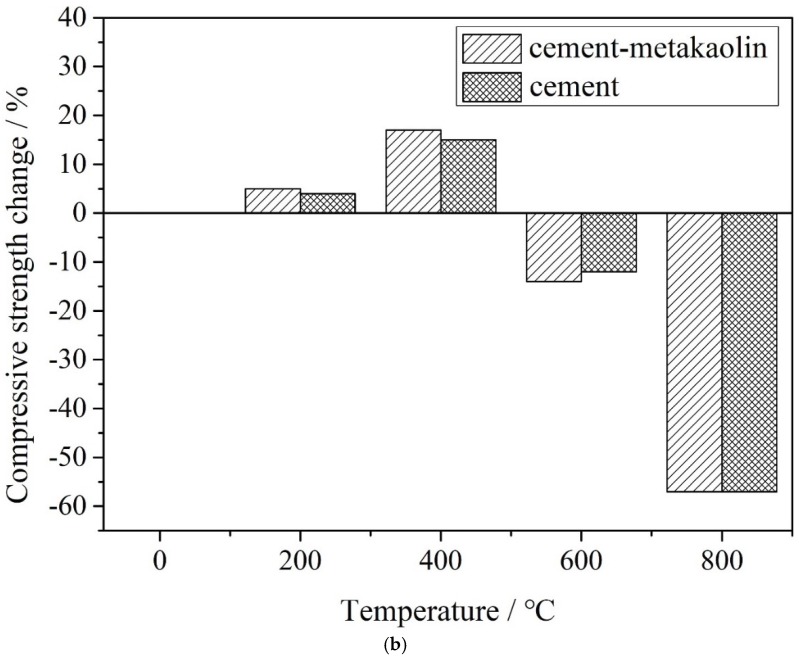
Effect of metakaolin on compressive strength of cement paste at different temperatures. (**a**) Compressive strength; (**b**) compressive strength change rate.

The effects of metakaolin addition and high temperature exposure on compressive strength of two groups of cement pastes are shown in Figure 3. Compared with strength obtained at ambient temperature, the compressive strength increases at 200 °C and 400 °C, respectively. However, up to 800 °C, the compressive strength decreases rapidly and notably. It can be observed that the MK addition has an improving effect on the compressive strength. Addition of 10% metakaolin causes an increase of about 15% on the compressive strength at room temperature. At different target temperatures, metakaolin addition enhances the compressive strength by about 18%, 23%, 10% and 5% at 200 °C, 400 °C, 600 °C and 800 °C, respectively. It is noted that the cement paste with MK has higher compressive strength compared with control specimen regardless of exposure temperature and compressive strength reaches the maximum value at 400 °C. Considering compressive strength evolution with temperature, MK leads to a poorer performance for T > 400 °C.

These results are consistent with that in previous research, which revealed the important role of metakaolin in cement or concrete. Rashad et al. [20] investigated the properties of the cement-GGBS paste modified with metakaolin subjected to elevated temperatures. The results showed that the compressive strength before and after being exposed to high temperatures increased with increasing MK content and the residual compressive strength also reached its maximum value at 400 °C.

Meanwhile, Bu et al. [21] carried out research work to assess the microstructure and strength of oil well cement blended with metakaolin, and they confirmed that metakaolin addition led to high strength curing at 150 °C and above regardless of the curing term. The cement pastes with addition of metakaolin presented high strength due to dense microstructure with low porosity and stable hydration products. Furthermore, metakaolin particles also resulted in low porosity of hardened pastes due to filling effect. 

Subasi et al. [22] also concluded that metakaolin replacement increased the strength due to the pozzolanic effect, which significantly increased the strength on the 56th and 90th days.

Based on the analysis of previously published work, the main reasons that determine the role of metakaolin in cement or concrete materials in terms of strength are: (1) the filling effect, and (2) the pozzolanic reactivity of metakaolin by consuming Ca(OH)_2_ for the formation of additional calcium silicate hydrate (CSH) [20,25,26,27]. The addition of metakaolin can improve the compactness of cement paste or concrete, which in turn improves the compressive strength.

**Figure 4:**
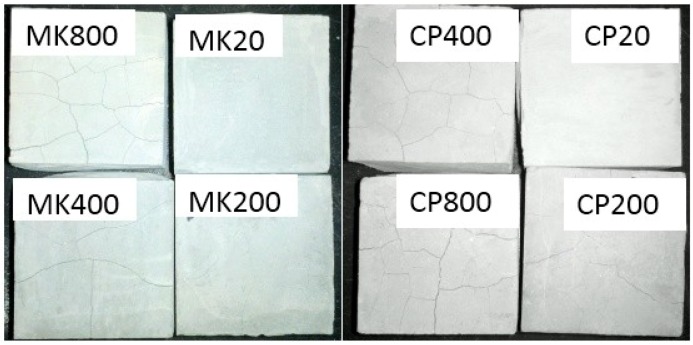
The appearances of cement–metakaolin paste and pure cement paste at different temperatures.

The typical appearances of the two series of pastes at ambient temperatures 200 °C, 400 °C and 800 °C are presented in Figure 4. It is indicated that the appearances for the two groups do not change obviously at ambient temperature and 200 °C. Only negligible cracks are observed on the surface of cement paste at 200 °C. However, when the temperature is higher than 400 °C, they are quite different from each other. The visible cracks begin to appear at 400 °C for both groups specimens and more cracks appear on surface of the pure cement group. The appearances at 800 °C are different, in which many cracks occur for the cement paste while it becomes relatively lesser for cement-metakaolin-blended paste. It does not seem easier for the crack to appear for the metakaolin group, which means the improvement effect of metakaolin and the relatively higher strength of cement–metakaolin paste as indicated in Figure 3. Pore connectivity and surface spalling phenomenon are also observed for both groups, which may mean the considerable drops of compressive strength as illustrated in Figure 3.

### 3.2. Pore Structure

**Figure 5:**
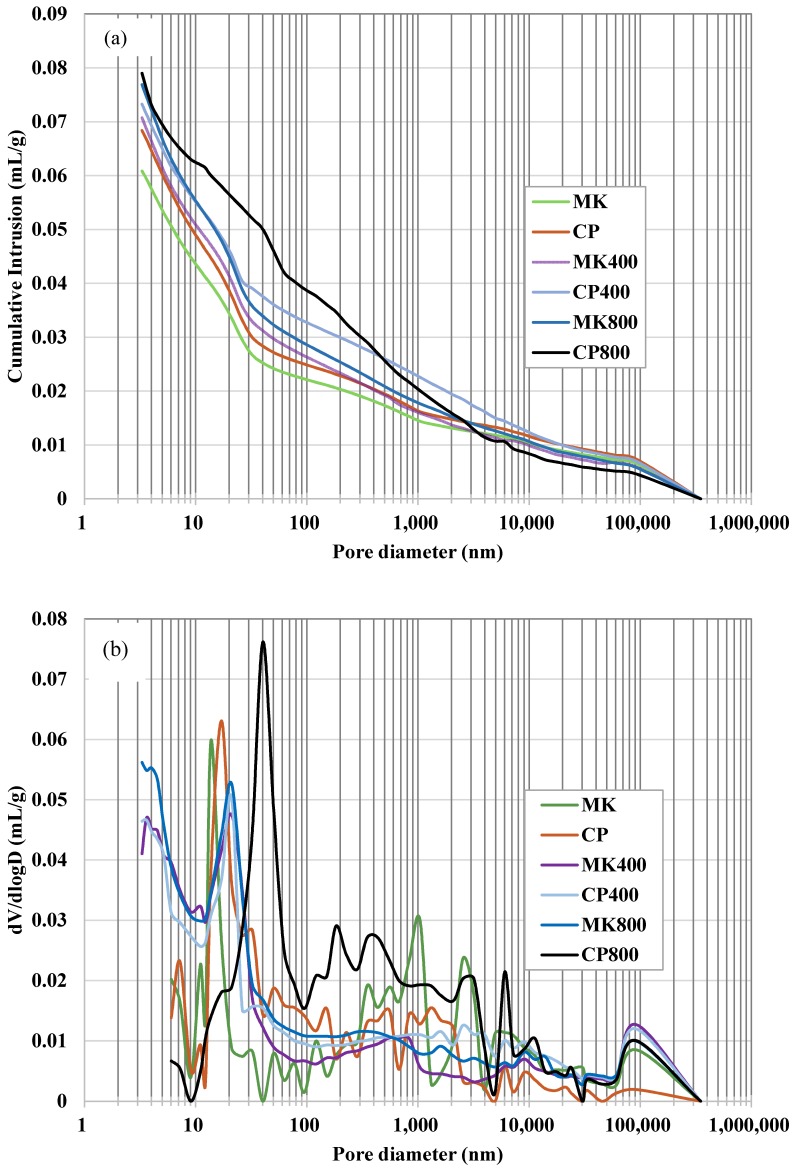
The porosity and pore size distribution of cement paste incorporating metakaolin before and after high-temperature exposure. (**a**) Cumulative intrusion; (**b**) incremental intrusion.

The pore structure including porosity and pore size distribution of cement pastes incorporating metakaolin before and after high temperature exposure were measured and evaluated by means of MIP and the results were provided in Figure 5. It could be clearly observed from Figure 5a that the cumulative intrusion volume related to the porosity decreases with the addition of metakaolin regardless of exposure temperatures. Furthermore, the proportion of pores with diameters larger than 10 nm increases after high temperature exposure and most of the pores are registered in the size ranges from 10 nm to 50 nm in diameter as shown in Figure 5b.

It could be clearly observed that the critical pore diameters, defined as the peaks in the differential curves, shift to low values with addition of metakaolin at different target temperatures. 

After thermal exposure, the peak values in the differential curves move to the pore areas with larger pore size when compared with room temperature group [10,28]. The thermal exposure degrades the pore structure. It is notable that the exposure of cement paste with or without metakaolin to elevated temperatures shifts the pore size peak and total porosity to higher values and also leads to the development of macropore system. 

The experimental results obtained from pore structure measurements correspond very well with the compressive strengths development as discussed above. The mechanical properties’ enhancement is attributed to the compact microstructure with low porosity [28].

### 3.3. Microstructure

**Figure 6:**
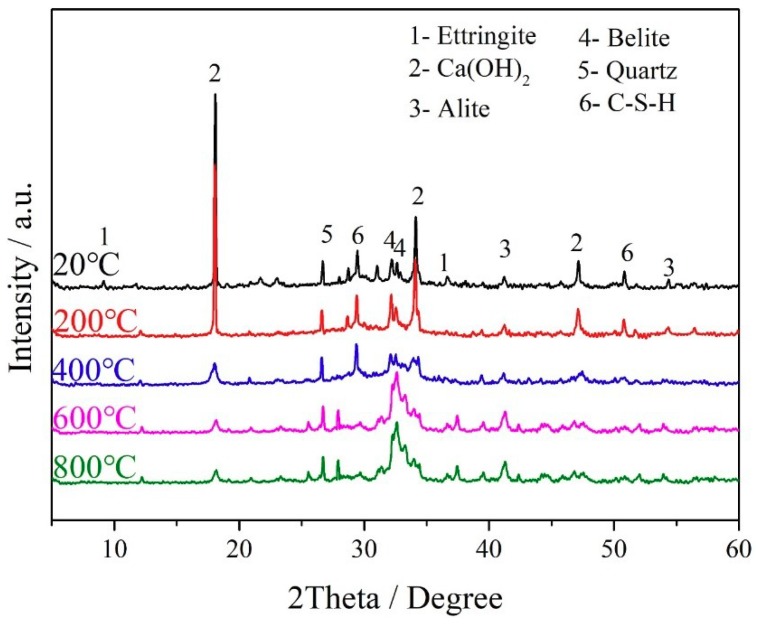
X-ray diffraction (XRD) patterns of cement–metakaolin pastes.

The XRD patterns of cement pastes blended with metakaolin at different temperatures were illustrated in Figure 6. The intensity of the peaks (2θ = 9.72°, 41.24°) decreased at 200 °C, indicating that most of the ettringite diminished at 200 °C [29]. Obviously, it could be judged from the attenuation of the peaks (2θ = 18.049°, 34.02°) at 400 °C. Moreover, C-S-H gel declined slowly as the temperature rose, to which the peaks related almost disappeared at 600 °C. Peaks related to the cement matrix (C_3_S and C_2_S) almost did not disappear at the whole heating process, which meant that the phase of the cement matrix did not change up to 800 °C [30]. 

**Figure 7:**
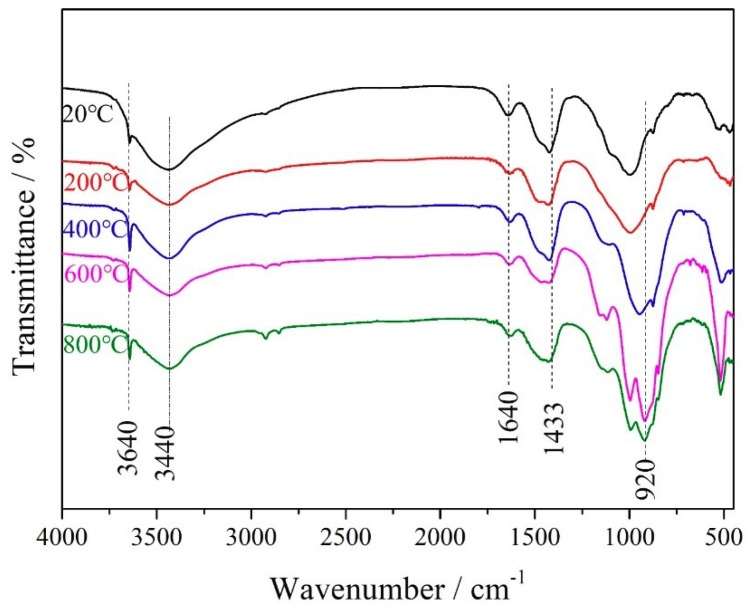
Fourier-transform infrared (FTIR) spectrum of cement-metakaolin pastes in KBr pellet.

As shown in Figure 7, the following bands could be observed on the FTIR spectra: calcium hydroxide band (3640 cm^−1^), carbonate phases (1433 cm^−1^), molecular water (3440 and 1640 cm^−1^). It could also be found that the band (3440 cm^−1^) combined and adsorbed water of C-S-H, AFm and AFt phases decreased with increasing temperature. The band (920 cm^−1^) appeared under the conditions of calcination at 600 °C and 800 °C, which proved the generation of anhydrous calcium silicates [31]. In addition, there was almost no difference in the FTIR spectra at 600 and 800 °C.

**Figure 8:**
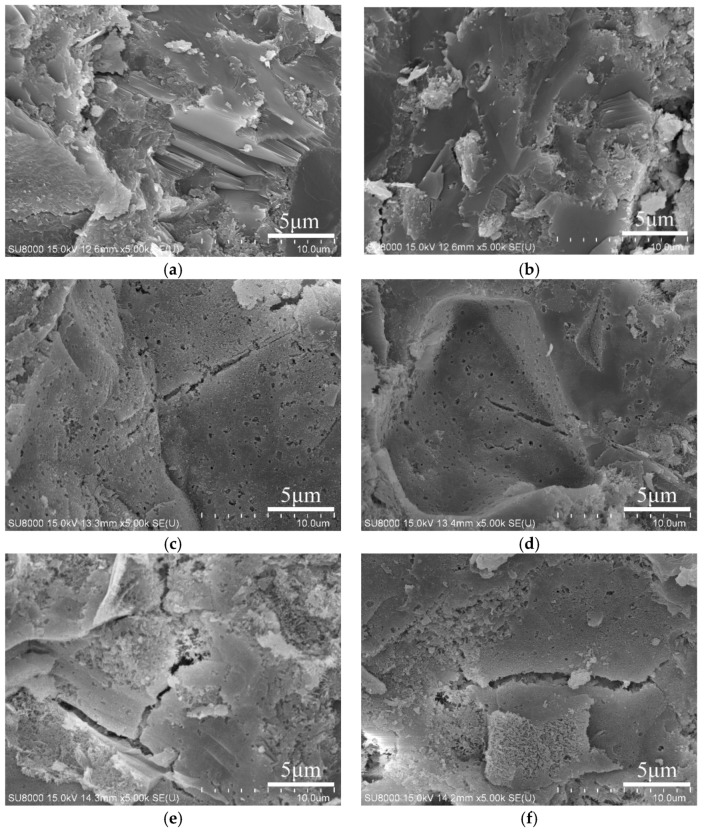
Micrographs of reference cement paste and 10% metakaolin-blended cement paste before and after 400 °C and 800 °C. (**a**) CP; (**b**) MK; (**c**) CP 400 °C; (**d**) MK 400 °C; (**e**) CP 800 °C; (**f**) MK 800 °C.

A SEM test were employed and carried out on paste specimens for the observation and identify the internal microstructure. The morphological changes of reference cement paste and 10% metakaolin-blended cement paste before and after 400 °C and 800 °C, respectively, were selected to assess the effects of metakaolin addition and high temperatures and the results were exhibited in Figure 8.

Products of hydration reaction of cement represent a heterogeneous material, i.e., the matrix comprising C-S-H and Ca(OH)_2_. The cement paste shows some cracks and a high ratio of porosity after 400 °C exposure due to the evaporation of water from C-S-H and the decomposition of portlandite (Ca(OH)_2_) [32,33,34]. With the increasing target temperature up to 800 °C, more porous structure could be observed compared to the reference paste due to the severe evaporation of water from C-S-H and the decomposition of portlandite (Ca(OH)_2_) [32,33,34]. 

Metakaolin addition results in a much denser microstructure before elevated temperatures exposure due to the pozzolanic reaction between metakaolin and cement hydration product Ca(OH)_2_, which leads to C-S-H gel formation and denser microstructure. Consequently, microstructure is transformed into a matrix associated with enhanced densification and little porosity. After thermal exposure at the target temperature of 400 °C, the microstructure of metakaolin-blended paste seemed to be slightly loose and Ca(OH)_2_ could not be found [34]. Increasing in temperature up to 800 °C leads to notable degradation in the microstructure, and high porosity and microcracks can be observed. The formations of microcracks or pores lead to further degradation in compressive strength in comparison with that obtained at the previous temperatures. However, 10% metakaolin addition still indicates good thermal resistance due to less cracks and relatively denser microstructure.

Based on the combination of the test results of compressive strength with microstructure from SEM as well as MIP as discussed above, it can be concluded that mechanical properties in the cement or blended cement pastes are closely related to microstructure development regardless of exposure temperatures.

### 3.4. Shrinkage

**Figure 9:**
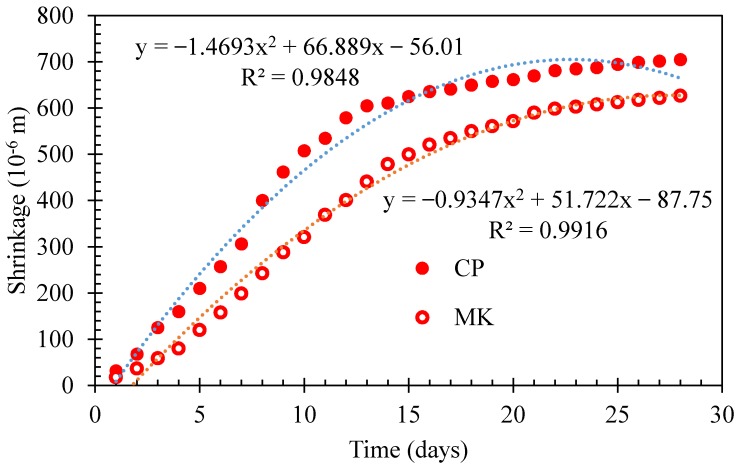
Shrinkage of cement paste and metakaolin-cement paste.

**Figure 10:**
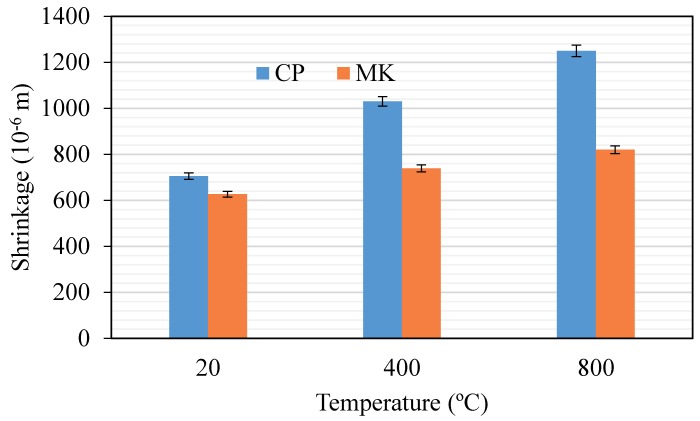
Effect of elevated temperatures on shrinkage of cement paste and metakaolin-cement paste.

Shrinkage results for the cement paste and metakaolin-blended paste specimens before and after thermal exposure have been illustrated in Figure 9 and Figure 10, respectively. All specimens were tested after pre-drying at 50 °C for 24 h to remove free water and also to avoid excessive shrinkage in the initial stage of the measurement and decomposition of hydration products.

Before high-temperature exposure, as indicated in Figure 9, all specimens exhibit shrinkage. Polynomial equation could be used to reveal the shrinkage evolution in cement paste or metakaolin-blended cement paste with testing days. The fitting coefficients (*R*^2^) are all excess 0.98 for the two series of paste. Obviously, cement exhibits higher shrinkage values when compared with that of metakaolin-blended pastes.

Figure 10 depicts results after 28 days. After being cured at a constant temperature for 27 days, a different series of paste specimens (20 °C group, 400 °C group and 800 °C group) with size of 20 × 20 × 200 mm^3^ were prepared for shrinkage test. Length changes of the specimens before and after thermal cycles were recorded and reported. The tests are carried out at the same age in non-heated and heated specimens. All specimens exposed to high temperatures are cooled naturally to room temperature, and the heating-cooling time span is 24 h, which is the same with the 20 °C group of specimens with 24 h of curing in a curing chamber with constant temperature of 20 ± 3 °C and relative humidity of 60 ± 5%.

After high temperature exposure, as indicated in Figure 10, compared to shrinkage at room temperature, cement paste and metakaolin-blended paste all exhibit higher shrinkage regardless of the target temperature. Cement still exhibits higher shrinkage values compared to that of metakaolin-blended pastes.

Based on the analysis of previously published work [35], the shrinkage of cement paste may be ascribed to a higher volume of mesopores, which causes a higher capillary stress by the water meniscus developed in the capillary pores of the paste, leading to a higher level of shrinkage [36].

These findings are compatible with the observations of previous researchers [37,38]. Gleize et al. [37] evaluated the influence of 5%–20% metakaolin addition on the autogenous shrinkage of cement pastes. They concluded that the long-term autogenous shrinkage of metakaolin-blended cement pastes decreased with increasing content of metakaolin, and autogenous shrinkage became lower for metakaolin-blended pastes compared to pure cement pastes due to the pozzolanic reactivity of metakaolin. Güneyisi et al. [38] found that the addition of metakaolin in cement played an important role and provided a great improvement to the pore structure and, therefore, metakaolin-modified cement or concrete had remarkably lower shrinkage strain than the control specimen.

### 3.5. Water Absorption

**Figure 11:**
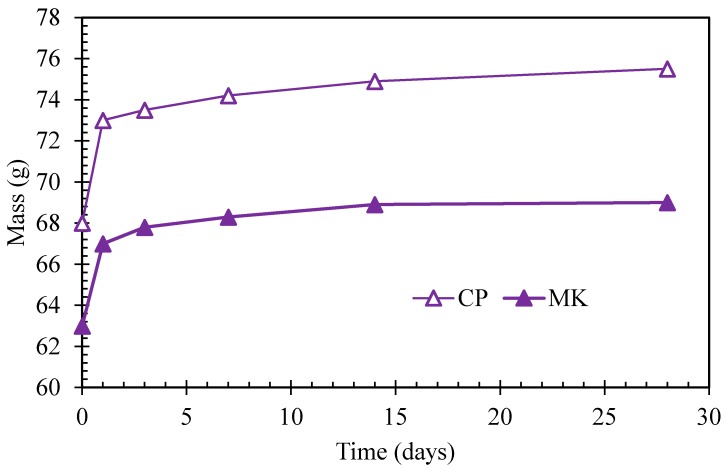
Mass change of cement paste and metakaolin-cement paste under the water absorption test before elevated temperature exposure.

Water absorption tests were conducted to determine mass change in the water absorption process of cement paste and metakaolin-blended paste, and the results before elevated temperature exposure have been presented in Figure 11. Metakaolin-blended paste exhibits lower water absorption compared to that of pure cement paste. Considering the pore structure results obtained by MIP as discussed above, the comparison between metakaolin-blended paste and neat cement paste shows that the total porosity decreases for blended pastes, which indicates that metakaolin-blended paste possesses denser microstructure. Denser microstructure relates to better anti-permeability, which means blended paste could withstand outside medium ingress including water and the conclusion is consistent with the water absorption test result. 

Before high-temperature exposure, cement paste and metakaolin-blended paste all exhibit evident water absorption compared to initial paste before being immersed in water, and the mass change tends to be slow with increasing immersion time.

**Figure 12:**
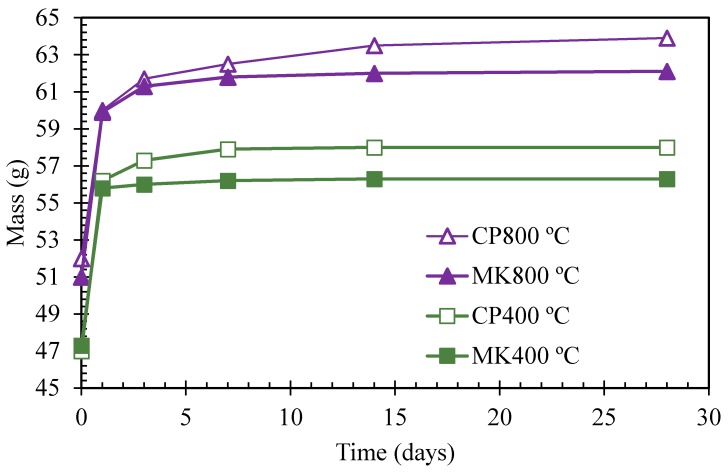
Mass change of cement paste and metakaolin-cement paste under the water absorption test after elevated temperature exposure.

**Figure 13:**
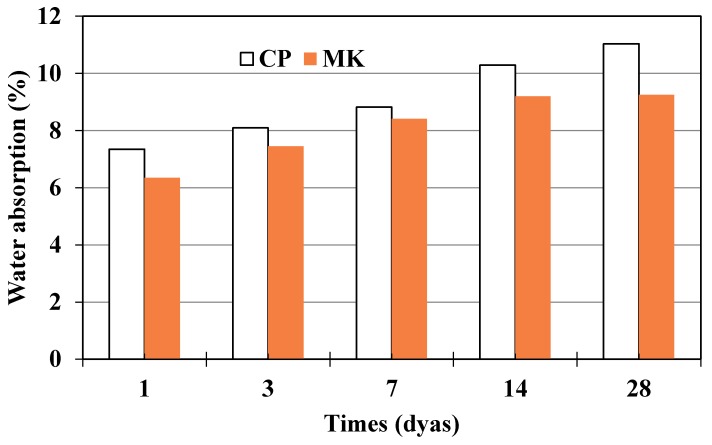
Water absorption of cement paste and metakaolin-cement paste.

**Figure 14:**
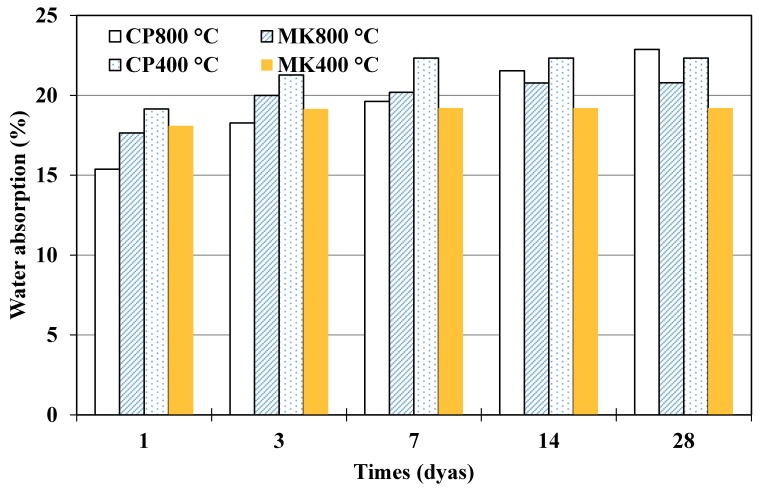
Water absorption of cement paste and metakaolin-cement paste after elevated temperature exposure.

The results of saturated water absorption on cement paste and metakaolin-blended specimens exposed to 400 °C and 800 °C have been presented in Figure 12. It indicates less water absorption in blended paste compared to pure cement paste. Water absorptions in % were also illustrated in Figure 13 and Figure 14. Comparison between Figure 11 and Figure 12 demonstrates that water absorption of specimens is relatively higher after being exposed to elevated temperatures. A denser microstructure relates to better anti-permeability, specimens after being exposed to 800 °C present looser microstructure than that of 400 °C group, which means outside medium such as water ingress easily, leading to higher water absorption.

## 4. Conclusions

This work presents the effects of 10% metakaolin addition on the compressive strength, water absorption resistance, shrinkage and microstructure evolution of cement paste. A comparison was made to evaluate the properties change before and after the elevated temperature exposure from room temperature to 800 °C. Based on the results of the laboratory tests, the following conclusions can be drawn.

(1) The compressive strength increases at 200 °C and 400 °C compared with that obtained at ambient temperature regardless of metakaolin addition. Up to 800 °C, the compressive strength decreases rapidly. The addition of 10% metakaolin causes an increase of about 15% on the compressive strength at ambient temperature and it enhances the compressive strength by about 20%, 24%, 14% and 15% at 200 °C, 400 °C, 600 °C and 800 °C, respectively. Compressive strength reaches the maximum value at 400 °C.
(2) The porosity decreases with the addition of metakaolin regardless of exposure temperatures and the critical pore diameters, defined as the peaks in the differential curves, shift to low values with the addition of metakaolin at different target temperatures. The thermal exposure degrades the pore structure and metakaolin addition improves the refinement of pore structure.
(3) The cement paste shows some cracks and a high ratio of porosity after 400 °C exposure. With the increasing target temperature to 800 °C, porous structure can be observed compared to the reference paste. Metakaolin addition results in much denser microstructure before and after elevated temperature exposure due to the pozzolanic reaction between metakaolin and hydration product CH, which leads to denser microstructure. Addition of 10% metakaolin presents good thermal resistance.
(4) As the temperature increases, the C-S-H gel in the cement paste dehydrates and forms anhydrous calcium silicate at 600 °C. The bonding between the hydration products is severely damaged, resulting in a sharp drop in the compressive strength of the cement paste.
(5) Polynomial equation can be used to reflect the shrinkage evolution in cement paste or metakaolin-blended cement paste with testing days. Cement exhibits higher shrinkage values compared to metakaolin-blended pastes regardless of the target temperature. After high temperature exposure, cement paste and metakaolin-blended paste all exhibit higher shrinkage compared to that at room temperature.
(6) The metakaolin-blended specimen indicates less water absorption compared to neat cement paste regardless of exposure temperature. Water absorption is relatively higher after exposed to elevated temperatures.

## Figures and Tables

**Table 1:** Chemical analysis and physical properties of ordinary Portland cement (OPC) and metakaolin (MK).

Item	OPC	MK
CaO (%)	68.30	0.04
SiO_2_ (%)	19.37	53.29
Al_2_O_3_ (%)	3.92	43.11
MgO (%)	1.61	0.22
Fe_2_O_3_ (%)	3.69	0.68
K_2_O (%)	0.59	0.42
Na_2_O (%)	0.13	0.44
SO_3_ (%)	0.81	0.11
TiO_2_ (%)	-	0.41
P_2_O_5_ (%)	-	0.35
Loss of ignition (%)	1.09	0.90
Specific gravity (kg/m^3^)	3110	2580
Specific surface (m^2^/kg)	369	12000

**Table 2:** Physical properties of cement.

Setting Time (min)		Flexural Strength (MPa)		Compressive Strength (MPa)	
Initial	Final	3 days	28 days	3 days	28 days
170	220	5.4	8.4	25.4	57.4

**Table 3:** Mix proportions of the cement paste. CP: Cement paste.

Specimens Code	Cement (g)	Metakaolin (g)	Water (g)	Superplasticizer (g)
**CP**	1000	0	400	10
**MK**	900	100	400	10
